# Koumiss (Fermented Mare’s Milk) as a Functional Food: Bioactive Proteins, Peptides, and Future Perspectives

**DOI:** 10.3390/foods14223954

**Published:** 2025-11-18

**Authors:** Borhan Shokrollahi, Jae-Young Choi, Miyoung Won, Eun-Tae Kim, Seung-Eun Lee, Jun-Sang Ham

**Affiliations:** Subtropical Livestock Research Center, National Institute of Animal Science, Rural Development Administration (RDA), Jeju 63242, Republic of Korea; borhansh@gmail.com (B.S.); mywon87@korea.kr (M.W.); etkim77@korea.kr (E.-T.K.); selee81@korea.kr (S.-E.L.)

**Keywords:** mare’s milk, koumiss, bioactive peptides, multi-omics, functional food

## Abstract

Fermented mare’s milk, or koumiss, has been consumed for centuries across Central Asia for its nutritional and therapeutic value. Mare’s milk differs from bovine milk by its near 1:1 casein-to-whey ratio, high lysozyme and lactoferrin, abundant immunoglobulins, and low β-lactoglobulin, which enhance digestibility, reduce allergenicity, and increase antimicrobial activity. During fermentation, lactic acid bacteria and yeasts transform this substrate into a reservoir of bioactive proteins, peptides, and metabolites. Multi-omics profiling has identified more than 2300 peptides and over 350 metabolites, including sequences with angiotensin-converting enzyme (ACE)-inhibitory, antioxidant, antimicrobial, and immunomodulatory activities. Preclinical and limited clinical data indicate potential benefits for lipid metabolism, cardiovascular function, and gut health. Mechanistically, these effects appear to arise from synergistic actions of native proteins, fermentation-derived peptides, and probiotic consortia. Technological advances such as rational starter culture design, controlled proteolysis, and microencapsulation offer strategies to enhance bioactive yield and stability. However, standardized fermentation protocols and clinical validation remain necessary to position koumiss as a scientifically supported functional food.

## 1. Introduction

Mare’s milk has been consumed across Central Asia for millennia, with archeozoological evidence from the Botai culture (~3500 BCE) confirming early horse domestication and milking practices [1,2,3,4]. Traditionally used fresh (*saumal*) or fermented as koumiss (*airag*, *qymyz*), it was prescribed in sanatoria across Russia and China for tuberculosis, anemia, and digestive disorders [5,6,7,8,9]. Today, an estimated 30 million people consume mare’s-milk products, with niche markets expanding into Europe [3,10,11,12].

Compositionally, mare’s milk more closely resembles human than bovine milk, with a protein balance that favors digestibility and peptide generation [4,13,14,15,16,17,18]. It is enriched with vitamins, minerals, amino acids, and polyunsaturated fatty acids and contains bioactive proteins such as lysozyme, lactoferrin, α-lactalbumin, and immunoglobulins, which contribute to antimicrobial and immune-supportive functions while reducing allergenicity [14,19,20,21,22,23].

Fermentation by lactic acid bacteria (LAB; mainly *Lactobacillus helveticus*, *L. plantarum*, *L. delbrueckii* subsp. *bulgaricus*, *Lactococcus lactis*, and *Streptococcus thermophilus*) and yeasts transforms mare’s milk into koumiss, a mildly alcoholic beverage (~0.6–3% *v*/*v*) enriched with lactic acid, CO_2_, and diverse metabolites [24]. Unlike conventional dairy fermentations that are mainly homolactic, koumiss undergoes dual lactic–alcoholic fermentation, where LAB and yeasts such as *Kluyveromyces marxianus* and *Saccharomyces cerevisiae* engage in mutualistic metabolism: LAB hydrolyze lactose and release amino acids that promote yeast growth, while yeasts regenerate cofactors and produce ethanol, CO_2_, and flavor-active volatiles [25,26,27]. This syntrophic system yields higher peptide and vitamin B diversity and unique sensory and bioactive properties compared with yogurt or kefir. Comparable dual fermentations using lactic acid bacteria and yeasts isolated from Korean dairy systems have been characterized for metabolic cross-feeding and bioactive formation. This process enhances microbial safety while expanding the pool of bioactive compounds that underpin its functional and therapeutic properties [28,29,30].

Peptidomic and metabolomic profiling confirm koumiss as a reservoir of functional molecules. More than 2300 peptides and over 350 metabolites have been identified, including sequences with antioxidant, antimicrobial, immunomodulatory, angiotensin-converting enzyme (ACE)-inhibitory, and dipeptidyl peptidase-IV (DPP-IV)-inhibitory activities [12,28,31,32,33,34,35,36,37]. A novel antimicrobial peptide (MP-4) showed potent activity against *Staphylococcus aureus* and stability across pH and temperature ranges [38,39]. Pathway-level shifts in amino acid and lipid metabolism further suggest mechanistic links to cardiovascular, digestive, and immune outcomes [40,41,42,43]. However, clinical evidence remains limited to small-scale or ethnomedical reports, and robust randomized controlled trials are lacking [7,44].

This review synthesizes current knowledge on bioactive proteins and peptides in fermented mare’s milk. It focuses on (i) compositional and nutritional features; (ii) native bioactive proteins such as lysozyme and lactoferrin; (iii) fermentation-derived peptides and mechanisms; (iv) technological strategies to enhance bioactivity; and (v) preclinical and clinical evidence. By integrating biochemical, microbiological, and nutritional perspectives, we highlight the unique functional value of fermented mare’s milk and its potential as a premium functional food.

### Literature Search Strategy and Review Framework

This review was developed using a structured synthesis approach consistent with PRISMA principles for scoping reviews. Relevant publications were retrieved from PubMed, Scopus, Web of Science, and Google Scholar, covering the period 2000–2025. Search terms combined “koumiss,” “mare’s milk,” “fermented mare milk,” “lactic acid bacteria,” “yeast fermentation,” “bioactive peptides,” “proteomics,” “peptidomics,” “metabolomics,” “functional foods,” “microbiome,” and “health benefits.”

Only peer-reviewed English-language articles were included. Non-research documents such as editorials, news items, or reviews lacking biochemical or microbiological data were excluded. Preference was given to studies reporting original findings on koumiss composition, microbial ecology, bioactive peptide or metabolite identification, and physiological or clinical outcomes.

All selected studies were critically assessed for methodological clarity, analytical reliability, and biological relevance. Data were synthesized to integrate compositional, mechanistic, and technological insights on koumiss fermentation and its translational potential as a functional food.

Furthermore, all cited preclinical and clinical studies were qualitatively evaluated for methodological rigor. Criteria included study design, presence of control groups, sample size, replication, and clarity of outcome measures. Randomized controlled trials and validated animal models were weighted more heavily than exploratory or in vitro studies. When data were limited or of low quality, this was explicitly noted in the text to ensure transparent interpretation.

## 2. Composition and Native Bioactive Proteins of Raw Mare’s Milk

Mare’s milk is distinct from ruminant milk yet closely resembles human milk in macronutrient profile, bioactive protein content and distinctive portfolio of bioactive proteins, which differ markedly from those of bovine milk. It contains approximately 2.1–2.7% protein, 1.0–1.5% fat, and 6–7% lactose, with a near-equal casein-to-whey ratio of approximately 1:1 [3,4,45,46]. In contrast, bovine milk averages 3.5% protein, 3.6% fat, and 4.6% lactose, with a casein-dominant profile (~80:20) [47]. Human milk, by comparison, contains ~1.0% protein, 3.8–4.0% fat, and ~7% lactose and is whey-predominant, shifting from ~90:10 in colostrum to ~60:40 in mature milk [48,49] (Table 1).

### 2.1. Casein and Whey Fractions

Mare’s milk proteins are distributed almost equally between caseins (approximately 49–53%) and whey (approximately 47–51%), yielding a ~1:1 ratio that contrasts with the casein-dominant bovine profile and aligns more closely with human milk [3,4,13,46]. Among caseins, β-casein and *α_s_*_1_-casein predominate, carrying multiple phosphorylation sites (2–8 for *α_s_*_1_-casein [54], 3–7 for β-casein [55]), which serve as proteolytic hotspots and release functional peptides during fermentation or digestion (see Section 4). *α_s_*_2_-casein occurs at much lower levels than in bovine milk, and together with the high whey proportion, it contributes to softer curd formation and faster gastric hydrolysis [56,57]. κ-Casein is particularly scarce; its first identification required specialized purification [58], and mare milk micelles, though relatively large (~255 nm), are less compact, further enhancing enzymatic accessibility and digestibility [57].

The whey fraction contains α-lactalbumin, serum albumin, lactoferrin, lysozyme, and immunoglobulins, with only trace levels of β-lactoglobulin compared with bovine milk [18,59]. α-lactalbumin–rich proteins can raise plasma tryptophan availability and, in vivo, have been associated with anxiolytic-like effects and improved sleep via serotonin pathways [60,61]. Lactoferrin and lysozyme exhibit potent antimicrobial and immunomodulatory effects demonstrated across animal models and clinical studies [61], and immunoglobulins contribute to passive mucosal defense and gut barrier protection [62,63]. Collectively, the balanced casein–whey distribution, micellar architecture, and extensive phosphorylation make mare’s milk an exceptional substrate for generating bioactive peptides with cardiometabolic, antimicrobial, and immunomodulatory functions [18,64].

### 2.2. Protein Composition and Key Bioactive Components

Lysozyme occurs at ~0.2–2 g/L, about tenfold higher than in bovine milk and slightly below human levels [53]. This elevated lysozyme enhances antimicrobial activity and supports gut microbial balance, resembling the protective effects of human milk [3,44]. Lactoferrin is present at ~0.08–0.20 g/L, higher than bovine but lower than human milk [65,66,67]; its greater abundance compared with cow milk confers stronger iron-chelating, antiviral, and immune-modulating functions, while still providing moderate antioxidant activity [68]. α-Lactalbumin (~1 g/L) is enriched relative to bovine milk, while immunoglobulins (IgG, IgA, IgM) are especially abundant in colostrum and decline during lactation [50,69,70]. By contrast, β-lactoglobulin, the principal bovine allergen, is substantially lower in mare’s milk (~2.55 g/L vs. ~3.2 g/L) [71,72,73]. Mare’s milk also contains endogenous enzymes such as lipase [74] and amylase [75], which may aid fat and carbohydrate digestion. These proteins and enzymes provide important precursors for bioactive peptides generated during fermentation, whose functional and mechanistic roles are discussed in Section 4.

### 2.3. Lipid Profile

Mare’s milk contains relatively little fat (~1.0–1.5%), much lower than bovine milk (3.5–4.0%) but similar to donkey milk (~1.0–1.8%) and considerably below human milk (~3.5–4.5%) [13,76]. Despite this, its lipid fraction is nutritionally significant due to its distinctive fatty acid composition and structural features.

Unsaturated fatty acids (UFA) constitute ~44–50% of total fatty acids, including 18–31% PUFA, with notable enrichment in linoleic (C18:2 n-6) and α-linolenic acid (C18:3 n-3) [77]. These levels are far higher than in bovine milk (3–5% PUFA) and goat milk (5–7%) and comparable to or greater than those in donkey milk (10–15%), though still lower than in human milk (15–20%) (Table 1). Mare’s milk also shows a favorable n-6/n-3 ratio, often as low as 1.5:1 in early lactation, superior to ruminant milks [3,4,7,78]. Elevated α-linolenic acid (~9–10% of total FA) and trace long-chain PUFA derivatives such as arachidonic acid and docosahexaenoic acid contribute to potential cardiovascular, anti-inflammatory, and neurodevelopmental benefits, although quantification of LC-PUFA remains limited [7].

Structurally, mare’s milk fat globules are smaller (2–3 µm) than those in goat or bovine (~4 µm) and comparable to those in donkey (2–3 µm) and human milk (3–4 µm), improving emulsification, gastric emptying, and lipase accessibility [13,77]. Comparative studies confirm a higher abundance of small fat globules (<5 µm) in horse and donkey milk, underpinning their higher digestibility [4,13,79]. Beyond fatty acids, mare’s milk contains phospholipids and sphingolipids, particularly phosphatidylethanolamine and phosphatidylserine, which play roles in membrane development, signal transduction, and inflammation modulation, though systematic quantification is scarce [3,53].

The lipid fraction also delivers fat-soluble vitamins (A, D_3_, E), present at levels comparable to bovine milk and proportionally enriched when normalized to fat content [3,4]. Overall, compared with ruminant milks, mare’s milk and other equid milks present a low-fat but highly unsaturated profile closer to human milk, supporting better digestibility and potential health benefits.

Milk composition varies with breed, lactation stage, and feeding system: pasture-based diets consistently improve PUFA levels and n-3 enrichment [3,46], while fatty acid proportions fluctuate from ~53–62% across breeds and lactation stages, underscoring the dynamic lipid profile of mare’s milk [80].

### 2.4. Carbohydrates and Oligosaccharides

The carbohydrate fraction of mare’s milk is dominated by lactose (~6–7%), a level substantially higher than bovine (~4.7–4.9%) and goat (~4.4%) milks and closely resembling human milk (~6.7%) [44,81]. This elevated lactose content imparts a sweet taste and improves palatability and digestibility, particularly in infants and individuals with compromised digestion [3].

Beyond lactose, mare’s milk contains a diverse oligosaccharide profile resembling human milk, with both sialylated and neutral structures. High-performance analyses have identified 3′-sialyllactose (3′SL), 6′-sialyllactose (6′SL), 3′-sialyl-N-acetyllactosamine (3′SLN), and sialyllacto-N-tetraose variants (LSTa–c) as major components [56]. Capillary electrophoresis studies reported high concentrations of 3′SL (~197 mg/L) and 6′SL (~82 mg/L), far exceeding those in donkey milk (~45–50 mg/L) [82]. These human-milk-like oligosaccharides (HMOs) are largely absent in bovine milk, making equid milks uniquely valuable among domestic species [83].

Mare’s-milk oligosaccharides (MMOs) display prebiotic activity, selectively stimulating beneficial gut microbes such as *Bifidobacterium* and *Lactobacillus*. They may also act as decoy receptors against enteric pathogens (e.g., *E. coli*, rotavirus) and contribute to immune modulation through cytokine regulation and T-cell responses [83,84,85,86,87]. Mare’s milk also provides N-acetylneuraminic acid (Neu5Ac, sialic acid) via its sialylated oligosaccharides, which supports neural development, synaptic plasticity, and antiviral defense [82].

Despite these promising features, the absolute concentrations, structural diversity, and in vivo significance of MMOs remain poorly characterized, largely due to limited sample sizes, breed variation, and the absence of standardized analytical protocols across laboratories. The analytical techniques used for oligosaccharide profiling (e.g., LC-MS, CE-LIF) are not yet standardized across laboratories, and longitudinal datasets tracking compositional changes throughout lactation are scarce [88]. Furthermore, most evidence for their prebiotic and immunomodulatory effects derives from in vitro or animal studies rather than controlled human trials, leaving their physiological relevance largely speculative. To advance this field, future research should prioritize (i) harmonized analytical protocols for MMO quantification, (ii) cross-breed and longitudinal sampling to assess natural variability, (iii) integrated metabolomic–microbiome studies linking MMO intake with gut microbial and immune outcomes, and (iv) well-designed human intervention trials to confirm their health-promoting effects and guide functional food development.

### 2.5. Enzymes and Micronutrients

Mare’s milk provides not only macronutrients and bioactive proteins but also a suite of enzymes and micronutrients that enhance its nutritional profile. Key endogenous enzymes include amylase, lipase, catalase, and peroxidase-like activity, which support digestibility and redox balance [44,89]. Although lactoperoxidase was initially assumed to be active, direct studies found no detectable activity [89,90]. Amylase facilitates starch hydrolysis, while lipase aids lipid digestion, complementing mare’s milk’s small fat globules and unsaturated fatty acid-rich profile [3,91]. A lactoperoxidase-like system, albeit at lower levels than in bovine milk, can generate antimicrobial radicals from SCN^−^ and H_2_O_2_, inhibiting microbial growth and extending shelf life [3,92].

Mare’s milk is also enriched in water-soluble vitamins (C, B_1_, B_2_, B_3_, B_6_, B_12_, folic acid) and lipid-soluble vitamins (A, D_3_, E, K_2_). Vitamin C is particularly elevated in mare’s milk, averaging 18–25 mg/L, compared with 1–2 mg/L in bovine milk, contributing to antioxidant capacity, while vitamin D and folate aid skeletal development and hematopoiesis [3,4,7,44].

Its mineral profile includes high levels of calcium, potassium, magnesium, and phosphorus, with trace iron, zinc, and copper [56,93]. Breed-specific studies (e.g., Basque Mountain Horses) confirm these trends and show that lactation stage and management affect mineral composition [94]. The Ca:P ratio (~1.6–1.8:1) closely matches that of human milk, which is favorable for bone mineralization [44]. Though iron is present at low levels, its partial binding to lactoferrin enhances bioavailability [95,96].

Enzymes and micronutrients act synergistically with proteins and lipids to enhance digestibility, redox balance, and immune support, reinforcing the overall nutritional value of mare’s milk [3,44,56]. Despite this richness, systematic datasets quantifying variations by breed, lactation stage, or diet remain scarce [46]. Thus, the micronutrient concentrations reported here represent general averages rather than breed-specific values. Moreover, the in vivo bioavailability of micronutrients and the precise contribution of enzymes like lipase and lactoperoxidase to human digestion and immunity are not yet fully understood.

### 2.6. Functional Significance

The compositional profile of mare’s milk distinguishes it from ruminant milks and aligns it more closely with human milk. Its near-equal casein–whey balance supports digestibility and gastrointestinal tolerance, while low β-lactoglobulin content contributes to reduced allergenicity [3,52,53,57,72,97,98]. Clinical reports indicate that mare’s milk is well tolerated by a majority of children with cow-milk protein allergy and by adults with lactose intolerance or mild atopic responses [19,72]. However, these findings largely derive from small, open-label, or observational studies lacking double-blind, placebo-controlled designs, and most have limited immunological profiling or long-term follow-up. Moreover, interindividual variability and potential cross-reactivity to casein fractions warrant caution before recommending mare’s milk as a universal alternative for allergic patients. Large-scale randomized clinical trials with standardized diagnostic criteria and mechanistic endpoints are needed to confirm its hypoallergenic potential and to define safe consumption thresholds.

Native bioactive proteins, including lysozyme, lactoferrin, α-lactalbumin, and immunoglobulins, contribute antimicrobial, antiviral, and immunomodulatory effects. Together with endogenous enzymes such as lipase and amylase, they enhance mucosal defense, regulate inflammation, and improve nutrient assimilation [46,56,67,99]. These proteins also provide precursors for functional peptides generated during fermentation (see Section 4).

The lipid fraction, though modest in total quantity, is notable for its enrichment in polyunsaturated fatty acids and a favorable n-6/n-3 ratio. Small fat globules improve emulsification and enzymatic access, while phospholipids and sphingolipids support membrane integrity and signaling processes [7,44,77,100,101,102]. Collectively, these attributes suggest anti-inflammatory, cardioprotective, and neurodevelopmental potential.

Carbohydrates, dominated by lactose and enriched in human-milk-like oligosaccharides, further enhance functional value. [103]. These oligosaccharides exhibit prebiotic activity by stimulating *Bifidobacterium* and *Lactobacillus*, reduce pathogen adhesion, and deliver sialic acid derivatives (Neu5Ac) important for cognitive development and antiviral defense [85,104,105,106,107]. In vivo evidence supports these functions: in a DSS-induced colitis mouse model, mare milk and fermented mare milk improved gut barrier integrity, modulated inflammatory cytokines, and restored microbial balance [108]. Animal models using mare’s milk have shown improved intestinal barrier integrity, enhanced sIgA levels, and favorable modulation of gut microbiota composition, with effects comparable to human milk [109].

Finally, endogenous enzymes (amylase, lipase, lactoperoxidase) and micronutrients (vitamins C, E, folate, calcium, magnesium, iron, zinc) complement these macronutrient features, strengthening digestive support, antioxidant defense, and bone health [26,27,28].

Taken together, the compositional and functional profile of mare’s milk strongly supports its classification as a naturally functional food and highlights its role as an ideal substrate for fermentation into koumiss, a product further enriched in bioactive peptides and metabolites.

## 3. Fermentation Ecology and Microbial Transformation in Koumiss

**Figure 1:**
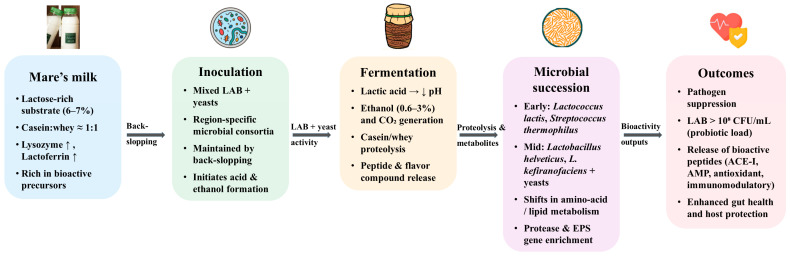
Koumiss fermentation process and microbial succession. Mare’s milk provides a lactose-rich substrate that supports lactic–alcoholic fermentation by LAB and yeast consortia. Microbial succession drives proteolysis and metabolite production, resulting in pathogen suppression, probiotic enrichment, and the release of bioactive peptides with antioxidant, antimicrobial, immunomodulatory, and antihypertensive functions. Arrows within the figure represent directional changes during fermentation: “↑” indicates an increase (e.g., lysozyme, lactoferrin, microbial load, metabolites), “↓” indicates a decrease (e.g., pH), and “→” denotes process progression from one stage to the next.

Koumiss (airag, qymyz), the traditional fermented mare’s milk of Central Asia, represents the biological interface between milk composition and microbial transformation. Consumed for centuries as both a food and a medicine, it has been credited with benefits ranging from improved digestion to adjuvant therapy for tuberculosis and anemia [9,24,75,110,111,112]. Its preparation relies on spontaneous lactic–alcoholic fermentation, in which consortia of LAB and yeasts transform the lactose-rich substrate into lactic acid, ethanol (0.6–3%), CO_2_, and a diverse spectrum of secondary metabolites [24,26,113,114]. Unlike bovine dairy fermentations, which typically employ well-defined starter cultures, koumiss depends on region-specific, mixed microbial communities maintained through back-slopping practices, yielding a product characterized by high microbial diversity and functional variability [25,112,114,115]. An overview of the fermentation process and microbial succession is illustrated in Figure 1. Comparative surveys across regions highlight both conserved LAB–yeast consortia and location-specific differences in koumiss microbiota (Table 2).

### 3.1. Core Microbial Groups

High-throughput sequencing consistently identifies *Lactobacillus helveticus*, *Lactococcus lactis*, *L. kefiranofaciens*, and *Streptococcus thermophilus* as dominant LAB [25,113,116]. Similar probiotic LAB from Korean raw-milk isolates exhibit antioxidant and biopreservative capacities [124,125], supporting the functional traits observed in koumiss communities. These species acidify the milk, suppress pathogens, and hydrolyze caseins and whey proteins, releasing bioactive peptides with antioxidant and antihypertensive potential [114,126]. *L. helveticus* is a notable source of ACE-inhibitory peptides such as Val-Pro-Pro (VPP) and Ile-Pro-Pro (IPP), while *L. kefiranofaciens* produces immunomodulatory exopolysaccharides (EPS) [35,127].

The yeast fraction, dominated by *Kluyveromyces marxianus* and *Saccharomyces cerevisiae*, drives the dual lactic–alcoholic fermentation typical of koumiss [25,128]. These yeasts metabolize sugars into ethanol and CO_2_, imparting effervescence, while producing esters, aldehydes, and other volatiles that shape flavor complexity [25,129]. *K. marxianus* is particularly versatile, generating ethanol, esters, and carboxylic acids that can be converted into flavor-active aldehydes, while also synthesizing B vitamins and antioxidant metabolites [130,131]. In addition to the core LAB and yeast species, the microbial composition of koumiss exhibits considerable regional and process-related variability. High-resolution sequencing studies have identified other lactic acid bacteria, including *Leuconostoc*, *Enterococcus*, and *Bifidobacterium*, as part of the microbial community, particularly in raw mare’s milk from different locations. Enterobacteriaceae such as *Acetobacter* spp. have also been detected, especially in samples from pastoral regions, indicating the influence of environmental and seasonal factors on fermentation dynamics [116].

Beyond these dominant groups, koumiss microbiota exhibits regional and environmental variability. Additional LAB such as *Leuconostoc*, *Enterococcus*, *Pediococcus*, and *Bifidobacterium* have been identified, particularly in raw mare’s milk or artisanal ferments [116,132,133,134]. *Leuconostoc* contributes diacetyl and acetoin for buttery flavor, *Pediococcus* enhances lactic acid production and probiotic functionality, and *Bifidobacterium* adds immunomodulatory potential. *Acetobacter* spp. have also been detected, especially in Russian koumiss, sometimes reaching ~18% of the microbial community [25,135,136]. Such diversity suggests that koumiss from different regions generates distinct repertoires of peptides and metabolites, contributing to variability in functional outcomes.

### 3.2. Effects on Safety and Probiotic Enrichment

Fermentation markedly improves the safety of mare’s milk. Raw milk may harbor opportunistic pathogens such as *Staphylococcus succinus*, *Klebsiella pneumoniae*, and *Acinetobacter lwoffii*, reflecting environmental exposure during milking and storage. These taxa are consistently absent in fermented koumiss, where rapid acidification (pH < 4.5), production of bacteriocins and hydrogen peroxide, and competitive exclusion by acid-tolerant LAB and yeasts suppress pathogens. High-throughput sequencing confirms their reduction within 24–48 h of fermentation, with microbial succession favoring LAB and ethanol-producing yeasts, thereby ensuring microbiological stability and extending shelf life under traditional and semi-industrial conditions [116,137].

Concurrently, koumiss becomes a dense reservoir of probiotics, with LAB populations typically reaching 10^8^–10^9^ CFU/mL and yeasts 10^6^–10^7^ CFU/mL [24,138]. Core LAB such as *Lactobacillus helveticus*, *L. kefiranofaciens*, and *Lactococcus lactis* exhibit probiotic functionalities including acid and bile tolerance, epithelial adhesion, and cytokine modulation [41,139,140,141]. Yeasts such as *Kluyveromyces marxianus* and *Saccharomyces cerevisiae* contribute by synthesizing B vitamins, antioxidant metabolites, and cell-wall polysaccharides with prebiotic activity. *K. marxianus* α-mannans serve as fermentable substrates that enhance short-chain fatty acid production and vitamin metabolism, while *S. cerevisiae* β-glucans and mannans add antioxidant and immunomodulatory effects [142,143].

Proteolytic LAB also generate bioactive peptides during fermentation. Casein- and whey-derived sequences, including β-casein fragments with ACE-inhibitory activity, have been identified (<3 kDa), with *L. helveticus* playing a key role [18,33,144]. These peptides act systemically after absorption, while live microbes exert local effects in the gut, providing a dual mechanism that may explain ethnomedical claims of koumiss as a remedy for digestive, respiratory, and immune-related conditions [24,110,145].

Despite promising findings, most evidence comes from small-scale in vitro or animal studies, and strain-level variability complicates generalization. Rigorous clinical trials and standardized fermentation protocols are needed to clarify koumiss’s safety mechanisms and probiotic potency in modern food systems.

## 4. Native Bioactive Proteins in Koumiss

**Figure 2:**
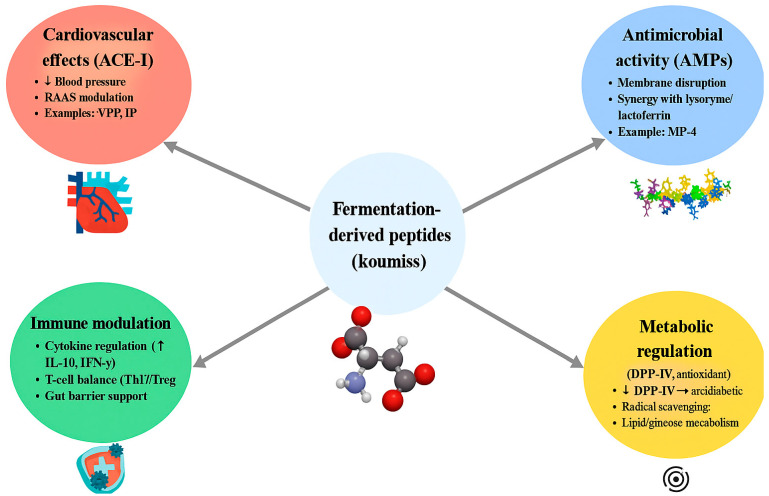
Mechanistic map linking peptide types in fermented mare’s milk to biological outcomes. ACE-inhibitory peptides act via the renin–angiotensin system; antimicrobial peptides disrupt microbial membranes; immunomodulatory peptides regulate cytokines and T-cell balance; DPP-IV inhibitory peptides target incretin pathways. Symbols in the figure denote directional changes: “↑” indicates an increase (e.g., cytokines IL-10 or IFN-γ), “↓” indicates a decrease (e.g., blood pressure or DPP-IV activity), and arrows show mechanistic links between peptide classes and their physiological effects.

As outlined in Section 2, mare’s milk contains a distinctive set of bioactive proteins, including lysozyme, lactoferrin, immunoglobulins, and α-lactalbumin, together with comparatively low levels of β-lactoglobulin [3,46,57]. Their concentrations and compositional comparisons with bovine and human milk have been detailed earlier; here, we focus on their functional and mechanistic roles. These proteins provide antimicrobial, antiviral, and immunomodulatory functions [46,56,67,99], and act as substrates for proteolysis during koumiss fermentation, generating peptides with cardiovascular, metabolic, and immune relevance [146,147,148]. The concentrations, functions, and peptide potential of the major proteins are summarized in Table 3. The mechanistic roles of these fermentation-derived peptides, including cardiovascular, antimicrobial, immunomodulatory, and metabolic pathways, are illustrated in Figure 2.

### 4.1. Lysozyme

Lysozyme is a hallmark protein of equid milk, present at ~0.25–0.50 g/L, compared with trace levels in bovine and caprine milk and similar to human levels [10,44,150]. Its abundance underpins the close functional resemblance between equid and human milks.

Functionally, lysozyme hydrolyzes the β-(1,4) glycosidic bonds in bacterial peptidoglycan, exerting strong antimicrobial activity against Gram-positive bacteria. Acting in synergy with lactoferrin, which permeabilizes Gram-negative membranes, lysozyme contributes to broad-spectrum antimicrobial defense [151,152]. Beyond its antimicrobial role, lysozyme contributes to gut homeostasis and immune development. It helps guide early microbial colonization and prevent dysbiosis, an effect particularly important for infants and immunocompromised individuals (Table 3). Beyond pathogen inhibition, lysozyme supports gut homeostasis and immune maturation. Supplementation studies in piglets and infants show stimulation of *Bifidobacterium* and *Lactobacillus* while suppressing harmful taxa, improving intestinal development and immune competence [153]. In murine models, oral lysozyme restored Paneth cell function, reduced intestinal permeability, and suppressed mucosal inflammation, highlighting its role in maintaining barrier integrity [154].

From an evolutionary perspective, lysozyme enrichment, especially high immediately after foaling, protects foals against enteric infection by shaping early microbial colonization and immune development [155]. Practically, this intrinsic antimicrobial capacity contributes to the natural preservation of raw mare’s milk, maintaining microbiological quality for up to 72 h under refrigeration [156].

Applied research extends these benefits to food systems. Equid milk’s heat-stable lysozyme improves product shelf life and bioactivity when retained during processing [19]. Extracted lysozyme from donkey milk has been tested as a natural preservative in yogurt, extending shelf life without altering sensory properties [157]. More broadly, lysozyme is under exploration for cheese production, biopreservation, and packaging films as a natural antimicrobial additive [152].

### 4.2. Lactoferrin

Lactoferrin concentrations in mare’s milk range from 0.08 to 0.20 g/L, exceeding bovine milk (0.02–0.10 g/L) but lower than human milk (1–3 g/L) [46]. As an iron-binding glycoprotein, lactoferrin contributes to host defense by sequestering free iron, destabilizing microbial membranes, and binding viral glycoproteins to block pathogen entry [158]. Proteomic studies confirm it as a major whey protein in mare’s milk, supporting its role in immune protection [3].

Equine lactoferrin also demonstrates antioxidant activity through free radical scavenging and metal chelation, enhancing neonatal defense against oxidative stress [159]. In vitro studies (mainly human or bovine lactoferrin) show activity against *Staphylococcus aureus*, *Escherichia coli*, and rotavirus [160,161], but equine-specific evidence is limited. In vivo, recombinant human lactoferrin (hrLF) reduced TNF-α expression during persistent reproductive inflammation in mares, highlighting immunomodulatory effects [162,163]. Lactoferrin functions synergistically with lysozyme (Section 4.1) to broaden antimicrobial activity [164]. Proteolysis of lactoferrin during fermentation or digestion releases potent peptides such as lactoferricin and lactoferrampin, which amplify antimicrobial and immunomodulatory activity beyond the intact protein [146,147,148]. Comparable milk-derived antihypertensive and antioxidant peptides have been characterized in Bifidobacterium and *Lactobacillus* fermentations of Korean milk [165,166], providing mechanistic parallels for koumiss peptide bioactivity. However, dose–response effects of equine lactoferrin in humans and the impact of fermentation on its stability in koumiss remain poorly characterized, representing key research gaps.

### 4.3. Immunoglobulins

Mare’s milk provides IgG, IgA, and IgM, supporting passive immunity in foals and mucosal defense in humans. In colostrum, IgG dominates (~30% of protein postpartum, declining to ~7% within 24 h) [18], while IgA is sustained during lactation, stabilizing after an initial decline and highlighting its role in mucosal protection [167]. IgG persists in mature milk, including in Chilean Corralero mares, suggesting ongoing immunological function [168]. Equine immunoglobulins neutralize pathogens, modulate cytokines, and interact with epithelial Fc receptors. Importantly, part of this activity is retained during fermentation, with IgG remaining detectable in koumiss [113]. Proteolysis may also yield immunoglobulin-derived peptides with antimicrobial or immunomodulatory potential, though these remain uncharacterized [169]. Thus, both intact immunoglobulins and their fragments likely contribute to koumiss’s immune-supportive properties.

### 4.4. α-Lactalbumin

α-Lactalbumin is a major whey protein in mare’s milk and the regulatory subunit of the lactose synthase complex [170]. It accounts for ~25% of whey proteins in late lactation [171] and is proportionally more abundant than in bovine milk, contributing to a human-milk-like whey profile [14,46,57]. Together with low β-lactoglobulin, this enhances digestibility and reduces allergenicity [3,50]. Rich in tryptophan and cysteine, α-lactalbumin supports serotonin synthesis, mood regulation, sleep quality, and glutathione-mediated antioxidant defense [172,173,174]. Structurally related to lysozyme (GH22 family), it also shows antimicrobial potential [170]. Bioactive derivatives include HAMLET (human α-lactalbumin made lethal to tumor cells), which induces apoptosis in tumor cells [175,176], and peptides with anxiolytic activity in rodent models [177]. Mare’s α-lactalbumin resists enzymatic hydrolysis, retaining activity through processing, and may generate additional bioactive peptides during fermentation, though these remain poorly defined [3,64].

### 4.5. β-Lactoglobulin and Allergenicity

Mare’s milk contains markedly lower levels of β-lactoglobulin (β-LG), the principal bovine whey allergen, reinforcing its hypoallergenic profile (see Section 2.1 and Section 2.6). Proteomic analyses confirm the relative scarcity of β-LG and enrichment of α-lactalbumin, immunoglobulins, and lysozymes compared with bovine and ovine milks [3]. Clinically, mare’s milk is well tolerated by most children with IgE-mediated cow’s milk protein allergy (CMPA), with rare sensitivities linked to heat-labile whey proteins [72]. As β-LG is absent from human milk, its reduced levels in mare’s milk strengthen its case as a CMPA-safe alternative [178]. Clinical trials in children confirm tolerance and nutritional adequacy of equid milks [72,179].

Fermentation further reduces β-LG immunoreactivity, suggesting koumiss may be even less allergenic [75]. The low abundance of β-LG also alters the proteolytic substrate pool, shaping the peptide repertoire of koumiss [15]. However, variability in β-LG quantification persists, and standardized assays are needed to validate safety across populations. Omics-based profiling and immunoassays will be critical to define how β-LG scarcity and fermentation influence both allergenicity and bioactive peptide generation.

## 5. Multi-Omics Insights and Technological Advances

**Figure 3:**
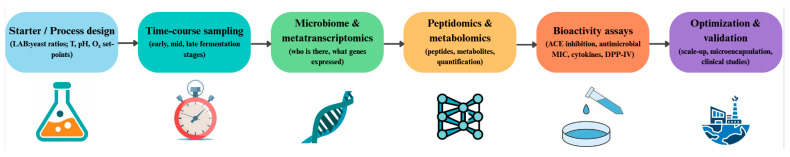
Omics-driven pipeline from koumiss fermentation to bioactive discovery. Integration of microbiome, peptidome, and metabolome profiling with bioactivity assays enables optimization of fermentation parameters, stability, and functional outcomes.

The application of multi-omics has shifted koumiss research from descriptive ethnomedicine to mechanistic understanding of how microbial consortia remodel mare’s milk into a matrix enriched in bioactive proteins, peptides, and metabolites. Integrated microbiome, proteomic/peptidomic, and metabolomic datasets are beginning to link specific microbial populations with functional output [180]. Similar HRMAS-NMR and MALDI-TOF metabolomic frameworks have been applied to characterize fermented dairy matrices in Korea, validating the multi-omics approach adopted here [181,182]. For instance, UPLC-Q-TOF-MS metabolomics reveals pathway-level shifts in amino acid and lipid metabolism between raw mare’s milk and koumiss, while peptidomics maps extensive proteolysis and peptide release. Long-read 16S and shotgun metagenomics resolve LAB–yeast community structures, reveal regional signatures, and identify functional gene clusters for proteolysis, amino acid transport, and bacteriocin biosynthesis [113,116]. Together, these approaches connect fermentation parameters (time, temperature, vessel type, starter ecology) with peptide release, metabolite shifts, and health-linked outcomes, laying the foundation for standardized, systems-level evaluation of koumiss [183] (Figure 3).

### 5.1. Metabolomics: System-Level Shifts During Fermentation

Untargeted metabolomics has cataloged ~354 metabolites in koumiss, with 61 significantly up-regulated and 105 down-regulated compared to raw mare’s milk [12]. Pathway enrichment highlights branched-chain amino acid metabolism, arginine–proline metabolism, and vascular smooth muscle contraction, suggesting possible cardiovascular relevance [12]. Fermentation elevates precursor amino acids (proline, valine, isoleucine), supporting microbial proteolysis and peptide formation. Koumiss contains ACE-inhibitory peptides derived from caseins and whey proteins [33], though specific motifs like Val-Pro-Pro and Ile-Pro-Pro remain to be confirmed. This metabolite–peptide axis illustrates how microbial metabolism drives functional bioactivity.

### 5.2. Proteomics and Peptidomics: Mapping the Peptide Repertoire

High-resolution LC–MS/MS reveals extensive proteolysis in koumiss, with >2300 peptides detected across mare’s milk and koumiss [28]. Earlier studies reported 24 peptides, primarily from β-casein and *α_s_*_1_-casein, with smaller contributions from κ- and *α_s_*_2_-caseins [15]. Functional assays demonstrate that peptides, particularly in the <3 kDa fraction, exhibit ACE-inhibitory, antimicrobial, and antioxidant activities, consistent with findings in other fermented milks [184]. In koumiss, the novel peptide MP-4 was identified with potent anti-*Staphylococcus aureus* activity [28]. Casein phosphopeptide hotspots and fragments of lactoferrin and α-lactalbumin also emerge as peptide precursors [18,185]. Time-resolved studies in dairy fermentations show that peptide abundance fluctuates, rising during active proteolysis and declining with prolonged maturation [186], a dynamic that warrants investigation in koumiss.

### 5.3. Microbiome and Metatranscriptomics: Who Is There and What They Do

Culture-independent surveys consistently show dominance of *Lactobacillus helveticus*, *Lactococcus lactis*, *L. kefiranofaciens*, and *Streptococcus thermophiles* [26,116]. In one PacBio study, *L. helveticus* accounted for 73.2% of reads, with *Lc. lactis* (7.3%), *L. kefiranofaciens* (6.1%), and *St. thermophilus* (4.0%) also prominent [116]. Metagenomics confirms LAB dominance (e.g., *L. helveticus* at ~79% across 23 samples) [26].

Yeast communities typically include *Kluyveromyces marxianus* and *Saccharomyces cerevisiae* [128], alongside taxa such as *Dekkera anomala*, *Kazachstania unispora*, *Meyerozyma caribbica*, *Pichia* spp., and others that enrich flavor complexity [25]. In vitro and metabolomic analyses have begun linking specific strains to functional outputs: *L. helveticus* and *L. kefiranofaciens* generate ACE-inhibitory peptides from casein substrate [25,187], while metabolomic work with *K. marxianus* supports enhanced metabolite production, including short-chain fatty acids, in fermented milk systems [188]. Functional metagenomics highlights enrichment of genes for proteases, amino acid transporters, glycoside hydrolases, and exopolysaccharide biosynthesis. Secondary metabolite clusters (lanthipeptides, bacteriocins, non-ribosomal peptides, polyketides) further emphasize koumiss as a reservoir of bioactive potential [189]. While most studies remain DNA-based, metatranscriptomics promises insight into which taxa are metabolically active. This could clarify contributions to amino acid catabolism, flavor compound synthesis, and EPS production, bridging presence and function [190]. Regional practices also shape microbial diversity: for example, koumiss produced in different containers (plastic, wood, leather) in Kyrgyz herds showed stable LAB cores but variable enrichment of *Bifidobacterium*, bacteriophages, and biosynthetic gene clusters, underscoring the ecological and functional diversity sustained by back-slopping traditions [113].

### 5.4. Microbiome and Metatranscriptomics: Who Is There and What They Do

Linking fermentation parameters with multi-omics data offers a pathway to optimize koumiss production through mechanistic insight. Fermentation duration strongly influences the biochemical profile of mare’s milk; one study reported peak nutrient levels at ~12 h [191], while broader dairy research shows metabolite changes occur most rapidly in early stages [192]. Vessel type also shapes functional diversity. Shotgun metagenomics of Kyrgyz koumiss found broadly similar microbial diversity across plastic, wooden, and leather containers, but plastic fermentations exhibited higher *Bifidobacterium* and bacteriophage levels, with genes for antimicrobial resistance and bioactive compound biosynthesis [113].

Other variables, including inoculum ratio, temperature, and vessel type, directly affect peptide and metabolite outputs. For example, a 1:1 LAB/yeast ratio optimized peptide release and sensory quality [138]. Integrated omics can link these variables to amino acid metabolism, protease gene expression, and bioactive peptide yield. Yeast–LAB co-metabolism further enhances amino acid availability, sustaining proteolysis and peptide generation [188]. Building on this, dual-omics frameworks propose coupling microbial community profiles with peptidomic readouts. Two-stage fermentations have demonstrated that aligning microbial succession with peptide release can maximize ACE-inhibitory activity while preserving sensory quality [193]. Such strategies parallel process-analytical technologies in industrial dairy systems and may provide a model for scaling koumiss while retaining functional benefits.

### 5.5. Technological Strategies to Steer Peptide Functionality

Several strategies aim to enhance peptide bioactivity and stability in koumiss. Rational starter design can pair complementary functions; for example, a proteolytic *Lactobacillus helveticus* with *Kluyveromyces marxianus*, which supplies flavor precursors and supports amino acid metabolism. Such co-cultures have been shown in milk systems to increase peptide diversity, flavor compounds, and ACE-inhibitory activity [194,195]. Protease control offers another lever. Selecting *L. helveticus* strains with robust cell-envelope proteinases (e.g., PrtH/PrtH2) and peptidases (PepN, PepX) or supplementing with exogenous proteases at controlled doses can enrich short bioactive peptides (<3 kDa). Over-hydrolysis risks bitterness, underscoring the need to balance enzyme activity, pH, and timing [196,197]. Reviews of dairy proteolysis provide guidance on strain selection and debittering strategies [193,198].

Stabilization technologies complement fermentation control. Microencapsulation with alginate (alone or layered with chitosan or gellan gum) improves LAB survival during storage and gastrointestinal transit, while freeze-drying or spray-drying with cryoprotectants (e.g., trehalose, skim milk) boosts viability of probiotics and peptide integrity. Recent optimizations using composite protectants and tuned drying conditions report substantial improvements in both probiotic survival and bioactivity [199,200,201].

### 5.6. Gaps, Standards, and Scale-Up Challenges

Despite advances, several limitations hinder the translation of koumiss from artisanal to industrial scale. The absence of standardized starter cultures contributes to variability in microbial composition, peptide yield, sensory quality, and ethanol levels across regions. Few studies systematically report fermentation parameters or peptide profiles, reducing reproducibility.

Scaling production raises further challenges: maintaining bioactivity and cultural authenticity while addressing regulatory issues (alcohol content, probiotic labeling, health claims) and ecological constraints of mare’s milk supply. Precision fermentation, quality-controlled starter banks, and process-analytical technologies could improve consistency, but successful adoption requires harmonized protocols, interdisciplinary research, and engagement with pastoral communities. Only by bridging traditional practices with modern standards can koumiss evolve into a reliable functional food without compromising its cultural heritage.

Although production of mare’s milk remains concentrated in Central Asia, notably Mongolia, Kazakhstan, Kyrgyzstan, Russia (e.g., Buryatia, Bashkortostan), and northern China, smaller-scale dairies and pilot programs are emerging in Europe (France, Italy, Germany) and North America [3]. In Kazakhstan, for example, studies report daily yields of ~5.7–7.7 L per day for Kazakh Jabe and Novo-Altay-Kazakh cross-bred mares over 105 days of lactation [202]. Against this backdrop, the global supply of mare’s milk remains very low compared with bovine milk, and production is highly seasonal and breed-specific. These constraints limit the industrial scalability of Koumiss and underscore the need for targeted breed improvement, standardized milking protocols, and geographically diversified sourcing to support global functional-food development.

## 6. Evidence in Animals and Humans

### 6.1. Insights from Animal Studies

Animal experiments consistently highlight the immunostimulatory and protective effects of koumiss. In cyclophosphamide-immunosuppressed rats, koumiss reversed thymus and spleen atrophy and increased leukocyte counts and CD4^+^/CD8^+^ ratios, indicating restoration of cell-mediated immunity [110]. In murine *Toxoplasma gondii* infection, koumiss shifted Th1/Th2 cytokines toward a balanced profile, reducing brain cyst burden during chronic infection [203]. Protective antioxidant and metabolic effects have also been documented. In rats co-exposed to mercuric chloride, probiotic koumiss reduced oxidative injury in the brain and kidney while normalizing GST and LDH [204]. Dried koumiss powder lowered serum cholesterol and triglycerides and increased leukocytes in rats [205], while koumiss supplementation in doxorubicin-treated rats reduced lipid peroxidation and restored antioxidant enzyme activity [206]. Casein-derived peptide fractions show in vitro radical scavenging, lipid protection, and metal chelation [207], supporting these in vivo outcomes.

Koumiss also mitigated acute alcohol intoxication in mice, improving behavioral and organ outcomes with supportive histology and transcriptomics [208]. In DSS-induced colitis, both mare’s milk and koumiss alleviated inflammation, improved colon structure, and modulated gut microbiota [108]. More broadly, fermented milks, including koumiss, enhance intestinal barrier integrity, increase short-chain fatty acids, and enrich *Lactobacillus* and *Bifidobacterium* [209]. While these findings are encouraging, most animal studies involve small cohorts and non-standardized koumiss preparations, limiting cross-study comparability. Larger, controlled models are needed to validate immune, antioxidant, and metabolic effects. An overview of representative animal and human studies is summarized in Table 4.

### 6.2. Evidence from Human Studies

Human data are limited but suggest cardiometabolic and gastrointestinal benefits. In hyperlipidemic adults, daily koumiss improved lipid profiles (lower total cholesterol, LDL-C; higher HDL-C), with metabolomics linking changes to gut-derived metabolites [26,211]. Small pilot studies hint at modest blood-pressure improvements [211], and metabolic profiling in healthy volunteers confirmed shifts in lipid and phospholipid pathways [210]. Koumiss contains in vitro ACE-inhibitory peptides [33]; analogous fermented milks enriched with these peptides have reduced blood pressure modestly in 4–8-week trials [212,213], suggesting a plausible but untested mechanism in koumiss.

Gastrointestinal improvements have been noted: in chronic atrophic gastritis, ~750 mL/day for 60 days reduced dyspeptic symptoms and increased fecal *Lactobacillus* abundance [43]. Broader reviews confirm fermented dairy products, including koumiss, support gut health through microbiota enrichment and SCFA production [209,214]

**Figure 4:**
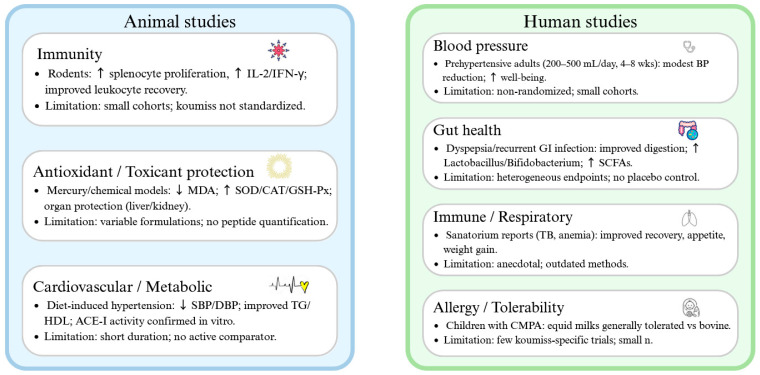
Evidence map of animal and human studies on koumiss. Animal studies demonstrate immunostimulatory, antioxidant, and cardiometabolic effects, while human reports suggest benefits in digestion, blood pressure, and allergy tolerance. However, most studies are limited in scale, duration, and standardization, underscoring the need for robust randomized clinical trials with peptide-level readouts. Symbols in the figure denote directional changes: “↑” indicates an increase (e.g., cytokines IL-2/IFN-γ, antioxidant enzymes, beneficial gut microbes), and “↓” indicates a decrease (e.g., MDA levels, systolic/diastolic blood pressure).

Historically, koumiss was prescribed in sanatoria across Russia and Mongolia for tuberculosis, anemia, and digestive disorders [75,215]. While these ethnomedical uses highlight a longstanding therapeutic reputation, modern randomized clinical validation is absent. More advanced are tolerability studies: in a controlled food challenge, mare’s milk was tolerated by 24/25 children with IgE-mediated cow’s milk protein allergy [72]. Donkey milk shows similar tolerability [216], underscoring the potential of equine milks as hypoallergenic alternatives, though no systematic koumiss trials exist. The distribution of evidence across animal and human studies, along with their endpoints and limitations, is summarized in Figure 4.

### 6.3. Mechanistic Considerations and Knowledge Gaps

Evidence from both animal and human studies suggests that koumiss exerts its effects through a combination of native mare’s-milk proteins and fermentation-derived bioactives. Mare’s milk is comparatively rich in lysozyme, lactoferrin, and immunoglobulins, providing innate antimicrobial and immune-modulatory capacity, while fermentation generates angiotensin-converting enzyme (ACE)-inhibitory peptides that could contribute to cardiometabolic effects [33,44]. Beyond simple compositional presence, studies on dairy peptides indicate that small di- and tripeptides can cross the intestinal epithelium via PepT1, and casein-derived tripeptides such as IPP and VPP have shown vascular effects in vivo, although robust pharmacokinetic data in humans remain limited and contested [217,218].

Critical knowledge gaps constrain translation. First, no randomized, placebo-controlled trials have systematically evaluated koumiss, and recent reviews of fermented foods stress the need for greater rigor and standardized reporting, with ISAPP consensus statements outlining best-practice trial design [219]. Second, dose–response relationships are undefined, preventing evidence-based consumption recommendations; by contrast, large epidemiological studies of other fermented dairy foods demonstrate progressive risk reductions for type 2 diabetes with higher intake, underscoring the importance of quantifying dose effects in koumiss [220]. Third, peptide pharmacokinetics and bioavailability remain poorly characterized in humans—although rapid absorption of di- and tripeptides is well established in animal and pig models, and vascular effects of IPP/VPP are documented, no data exist for koumiss-derived sequences [221,222]. Finally, reproducibility is hampered by incomplete reporting of koumiss composition; many studies omit basic details such as microbial profiles, peptide repertoires, or ethanol content, despite evidence that alcohol concentrations vary widely (≈0.6–3% depending on yeast activity, vessel, and season), complicating interpretation and raising safety and regulatory considerations. This highlights the need for standardized characterization of koumiss preparations in future clinical research [15,24,114,138].

## 7. Future Perspectives

Despite advances in understanding the functional properties of fermented mare’s milk, particularly its native proteins and fermentation-derived peptides, several research frontiers remain untapped. Although koumiss contains ACE-inhibitory peptides, their physiological relevance remains largely theoretical without targeted studies of in vivo pharmacokinetics and dose–response effects. Establishing causal links between peptide exposure and outcomes such as blood pressure reduction or immune modulation will require carefully controlled animal models and human trials that measure both functional endpoints and circulating peptide levels [33,206]. Applied with standardized protocols and shared in open-access repositories, these methods can elucidate organism–function relationships and improve reproducibility across geographic and production contexts [223,224].

The absence of well-designed randomized controlled trials remains a major barrier to translation. Future studies should rigorously characterize koumiss in terms of starter strains, fermentation parameters, peptide and metabolite profiles, and ethanol content, while incorporating robust biomarkers such as blood pressure, vascular reactivity, immune indices, and targeted peptide pharmacokinetics [220]. To achieve the greatest impact in advancing koumiss as a functional food, research priorities should focus on in vivo validation of peptide bioactivity, integration of multi-omics to map microbe–metabolite interactions, and optimization of fermentation processes that link microbial composition to reproducible biofunctional outcomes.

The lack of standardized starter cultures continues to drive variability in microbial composition, peptide yield, and sensory quality, undermining both reproducibility and industrial scalability. Although precision fermentation, rational starter design, and process control could enable consistent production of peptide-enriched koumiss with reliable bioactivity [9,19], practical challenges remain substantial. These include the difficulty of maintaining synergistic microbial consortia outside traditional back-slopping systems, variation in mare-milk composition and seasonal availability, and limited access to fermentation infrastructure in pastoral regions. Addressing these barriers will require collaborative pilot-scale trials and microbial selection strategies that preserve desirable strains while ensuring product consistency.

Finally, scaling production for functional food applications must balance ecological sustainability, economic feasibility, and cultural viability. Mare’s milk is seasonal and low-yield, and koumiss remains deeply embedded in the pastoral traditions of Central Asia and Mongolia. Future innovation must therefore reconcile cultural preservation and rural livelihoods with the development of koumiss as a standardized, globally distributed functional food [9,225]. Co-development frameworks involving local producers, cooperatives, and research institutions combined with fair-trade and geographical-indication models can ensure cultural authenticity while enabling equitable economic participation.

## 8. Conclusions

Koumiss represents a unique functional food that combines the intrinsic bioactivity of native proteins with a diverse repertoire of fermentation-derived peptides and metabolites. Evidence from biochemical, microbiological, and omics studies underscores its potential in immune modulation, cardiovascular support, metabolic regulation, and gut health, with reduced allergenicity compared to bovine milk. While promising preclinical and limited clinical findings exist, the absence of standardized fermentation protocols, dose–response data, and rigorous randomized trials constrains translation into mainstream applications. Future research integrating omics-driven characterization with well-designed clinical studies will be critical to validate health claims and enable the development of consistent, peptide-enriched koumiss products that honor traditional practices while expanding global functional food markets.

## Figures and Tables

**Table 1:** Comparative composition of mare’s, bovine, goat, donkey, and human milk (per 100 mL or as indicated).

Component	Mare *	Cow *	Goat *	Donkey *	Human *	Functional Note	References
Protein (%)	2.1–2.7	3.2–3.5	3.0–3.5	1.6–1.8	1.0–1.2	Lower in mare/donkey → closer to human milk, easier digestibility	[13,50]
Fat (%)	1.0–1.5	3.5–4.0	3.8–4.5	0.3–1.8	3.5–4.5	Mare’s milk is low-fat, hypoenergetic; donkey similar	[13]
Lactose (%)	6.0–7.0	4.6–4.8	4.5–4.7	6.2–7.0	6.5–7.0	High lactose enhances digestibility and prebiotic effect	[13,50]
Casein/whey ratio	~1:1	80:20	75:25	~50:50	~40:60	Mare/donkey closer to human → digestibility, peptide precursors	[3,44]
β-casein	High; multiple phosphorylation sites	High	High	Moderate	High	Precursors for ACE-I peptides	[16]
*α_s_*_1_-casein	Low	High	High	Low	Low	Low *α_s_*_1_-CN reduces allergenicity	[16]
β-lactoglobulin (g/L)	Very low	3–4	3–4	Trtableace	Absent	β-LG is main bovine allergen; low in mare’s milk reduces CMA risk	[3,20,51]
α-lactalbumin (g/L)	~1.0	1.5	1.3	1.0–1.2	1.2	Lactose synthesis; antioxidant, antimicrobial peptides	[50,52]
Lysozyme (g/L)	0.25–0.50	Trace	Trace	1.0–1.5	0.1–0.3	Strong antimicrobial, gut microbiota modulation	[3,13]
Lactoferrin (g/L)	0.08–0.20	0.02–0.1	0.02–0.1	0.1–0.3	1–3	Antimicrobial, antiviral, immune modulation	[3,13]
Immunoglobulins (g/L)	0.7–1.2 early lactation	0.6–0.8	0.6–0.9	0.5–1.0	0.5–1.0	Passive immunity, mucosal defense	[19]
Fatty acids (% PUFA)	18–31% total FA; α-linolenic acid enriched	3–5%	5–7%	10–15%	15–20%	PUFA content supports cardiovascular health	[3,13,50]
Fat globule size (µm)	2–3	~4	~4	2–3	3–4	Smaller globules aid digestion	[13,53]
Oligosaccharides	6–7% lactose; sialylated OS (3′SL, 6′SL, LSTa–c)	4.6% lactose	4.5%	6–7% + HMO-like OS	Rich in HMOs	Prebiotic and immunomodulatory potential	[3,13]
Ca:P ratio	1.6–1.8:1	1.3:1	1.3:1	1.5:1	1.6–2.0:1	Favorable for bone development	[13,50]

* Values represent average or general ranges compiled from multiple studies across breeds and lactation stages; minor variations may occur depending on environmental and nutritional factors.

**Table 2:** Koumiss microbial communities across regions.

Region	Dominant LAB	Dominant Yeasts	Other Taxa Reported	Functional Traits	References
Inner Mongolia/Xinjiang (China)	*Lactobacillus helveticus*, *Lactococcus lactis*, *Streptococcus thermophilus*	*Kluyveromyces marxianus*, *Saccharomyces cerevisiae*	*Leuconostoc mesenteroides*	Acidification, pathogen suppression, ACE-I peptide generation	[116,117]
Kazakhstan	*Lactobacillus kefiranofaciens*, *L. helveticus*	*Saccharomyces cerevisiae*	*Pediococcus acidilactici*	Exopolysaccharide production, antimicrobial peptides	[118,119]
Kyrgyzstan (high pastures)	*Lactobacillus delbrueckii ssp. bulgaricus*, *Lactococcus lactis*	*Kluyveromyces marxianus*	*Acinetobacter*, *Staphylococcus* (suppressed post-fermentation)	Proteolysis, pathogen inhibition	[25,120]
Russia (Buryatia, Altai)	*Lactobacillus helveticus*, *Lactococcus lactis*	*Candida kefyr*, *Kluyveromyces marxianus*	*Enterococcus faecium*	Aroma, antimicrobial activity	[21,121]
Europe (experimental koumiss)	*Lactobacillus plantarum*, *L. casei*	*Saccharomyces cerevisiae*	*Bifidobacterium breve* (starter adjunct)	Probiotic enrichment, DPP-IV inhibition	[122,123]

**Table 3:** Native bioactive proteins in mare’s milk: concentrations, functions, peptide potential, and evidence strength.

Protein	Concentration in Mare’s Milk *	Comparative Levels	Primary Functions	Peptide Potential	Evidence Strength (Refs)
Lysozyme	~99 mg/L (0.099 g/L)	~5× higher than human milk (~21 mg/L); trace in bovine	Antimicrobial (especially Gram-positive), immune modulation	Stable across fermentation; fragments likely antimicrobial	Strong—compositional and functional data [44]
Lactoferrin	~80–218 mg/L (0.08–0.22 g/L)	Higher than bovine; lower than human milk (~1–3 g/L)	Antimicrobial, antiviral, immunomodulatory, iron sequestration	Precursors to lactoferricin/lactoferrampin peptides	Moderate—quantified values from mare milk [95]
Immunoglobulins (IgG, IgA, IgM)	~0.88 g/L IgG at 0–12 h postpartum; plus IgA (~36.5 g/L), IgM (~14.1 g/L) [149]	Higher than typical cow milk (not quantified), lower than colostrum	Passive and mucosal immunity, cytokine modulation	Fermentation-derived peptides possible but uncharacterized	Moderate—based on temporal Ig quantifications [149]
α-Lactalbumin (α-LA)	Qualitatively abundant; similar to human milk and higher than bovine, exact g/L not provided	Higher than cow, similar to human milk [3,50]	Involved in lactose synthesis, antioxidant, immunomodulatory	Digestion releases antimicrobial and bioactive peptides	Moderate—qualitative abundance supported [3,50]
β-Lactoglobulin (β-LG)	Very low/reduced levels (specific values not provided)	High in bovine (~3–4 g/L), absent in human milk	Major bovine allergen; low levels reduce allergenicity	Low abundance limits peptide generation during fermentation	Moderate—clinical tolerance literature [53]

* Values represent average or general ranges compiled from multiple studies across breeds and lactation stages; minor variations may occur depending on environmental and nutritional factors.

**Table 4:** Representative animal and human studies on fermented mare’s milk (koumiss): models, endpoints, and outcomes.

Study Type	Model/Population	Intervention	Endpoints	Key Findings	Limitations	Reference
Animal—immunity	Immunosuppressed rats (cyclophosphamide)	Koumiss	Spleen/thymus index; leukocytes, lymphocytes, CD4+/CD8+ ratio; Peyer’s patches	Improved immune organs and lymphocyte recovery	Limited sample sizes; composition undefined	[110]
Animal—cardiovascular/metabolic	Hyperlipidemic models	Koumiss or fermented mare’s milk	Lipid profiles; ACE-inhibitory activity	Lipid metabolism and ACE-I activity—supported by peptidomic data	No clinical comparator; short duration	[210]
Animal—anti-inflammatory/gut health	Mouse ulcerative colitis model	Fermented mare’s milk	Colitis scores, inflammation markers	Reduced colitis inflammation, modulated flora	Preclinical; specific peptides not profiled	[108]
Human—allergy/tolerability	(Some studies exist, but koumiss-specific small)	Equid milk intake	Tolerance, allergic reactions	Mare/donkey milk generally tolerated in CMA patients	Small numbers; not koumiss-specific in all; more data needed	[75]

## Data Availability

No new data were created or analyzed in this study. Data sharing is not applicable to this article.

## Abbreviations

The following abbreviations are used in this manuscript:

ACE	Angiotensin-Converting Enzyme
CMPA	Cow’s Milk Protein Allergy
CO_2_	Carbon Dioxide
DPP-IV	Dipeptidyl Peptidase-IV
EPS	Exopolysaccharides
FA	Fatty Acids
GST	Glutathione S-Transferase
HAMLET	Human α-Lactalbumin Made Lethal to Tumor Cells
HDL-C	High-Density Lipoprotein Cholesterol
HMOs	Human Milk Oligosaccharides
IPP	Isoleucine-Proline-Proline
LAB	Lactic Acid Bacteria
LC-PUFA	Long-Chain Polyunsaturated Fatty Acids
LDL-C	Low-Density Lipoprotein Cholesterol
MMOs	Mare’s Milk Oligosaccharides
Neu5Ac	N-Acetylneuraminic Acid
PepT1	Peptide Transporter 1
PUFA	Polyunsaturated Fatty Acids
SCFA	Short-Chain Fatty Acids
TNF-α	Tumor Necrosis Factor Alpha
UPLC-Q-TOF-MS	Ultra-Performance Liquid Chromatography–Quadrupole Time-of-Flight Mass Spectrometry
VPP	Valine-Proline-Proline
